# Draft Genome Sequences of 10 *Microbacterium* spp., with Emphasis on Heavy Metal-Contaminated Environments

**DOI:** 10.1128/genomeA.00432-15

**Published:** 2015-05-14

**Authors:** Erika Corretto, Livio Antonielli, Angela Sessitsch, Petra Kidd, Nele Weyens, Günter Brader

**Affiliations:** a Health and Environment Department, Bioresources Unit, AIT Austrian Institute of Technology, Tulln an der Donau, Austria; b Instituto de Investigaciones Agrobiológicas de Galicia (IIAG), Consejo Superior de Investigaciones Científicas (CSIC), Santiago de Compostela, Spain; c Centre for Environmental Sciences, Hasselt University, Diepenbeek, Belgium

## Abstract

*Microbacterium* spp. isolated from heavy metal (HM)-contaminated environments (soil and plants) can play a role in mobilization processes and in the phytoextraction of HM. Here, we report the whole-genome sequences and annotation of 10 *Microbacterium* spp. isolated from both HM-contaminated and -noncontaminated compartments.

## GENOME ANNOUNCEMENT

*Microbacterium* spp. have been isolated from diverse environments, including soil, plants, water, and humans (1–4). They belong to the class *Actinobacteria*, known for the production of a rich spectrum of secondary metabolites, including organic acids, antibiotics, siderophores, and other chelators (5). Interestingly, several *Microbacterium* spp. have shown to be resistant to different HM and to influence the mobility of HM in contaminated soils (6–8). The influence on HM mobilization might be due to the secretion of specific metabolites, making these bacteria suitable candidates for not only the understanding of metal mobilization processes but also the improvement of phytoextraction techniques limited mainly by the plant biomass production and the rate of metal uptake (9, 10). Bacteria isolated from HM-contaminated environments can overcome these limitations by promoting plant growth and increasing HM availability. Therefore, we decided to sequence a set of *Microbacterium* sp. genomes of organisms isolated from different HM-contaminated sites in Europe and strains obtained from the DSMZ collection (http://www.dsmz.de/catalogues/catalogue-microorganisms.html). Based on related publications (11–15), we conclude that the DSMZ strains do not derive from HM-polluted environments (Table 1).

**TABLE 1:** Summary of isolate origins and genomic features

*Microbacterium* sp.^a^	Isolate	Source	Viral sequence % estimated with PhyloSift	No. of rRNAs in Barrnap/total no.^b^	No. of tRNAs	No. of ORFs^c^	G+C content (%)	*L*_50_^d^	*N*_50_^d^	No. of contigs	Total bp	Coverage (×)	Accession no.
From HM noncontaminated environment													
*M. azadirachtae* NR_116502	DSM 23848	Rhizoplane of neem (*Azadirachta indica*) seedlings	28^e^	3/12	49	3,751	70.45	12	97,758	86	4,037,586	49.69 ± 16.7	JYIT00000000
*M. foliorum* NR_025368	DSM 12966	Phyllosphere of grasses	10	5/11	47	3,324	68.7	6	151,883	46	3,558,318	74.75 ± 24.7	JYIU00000000
*M. ginsengisoli* NR_041516	DSM 18659	Soil of ginseng field	24^e^	3/3	45	2,963	70.22	8	145,068	80	3,047,504	71.53 ± 29.4	JYIY00000000
*M. ketosireducens* NR_024638	DSM 12510	Soil	26	3/12	49	3,481	70.27	10	115,384	57	3,922,598	56.55 ± 21.1	JYIZ00000000
*M. trichothecenolyticum* NR_044937	DSM 8608	Soil	23^e^	3/15	48	4,074	70.16	8	220,592	41	4,524,308	132.14 ± 42.4	JYJA00000000
From HM contaminated environment													
*M. oxydans* JX185498	BEL4b	Rhizosphere of *Brassica napus* (Lommel Field, Belgium)	25	3/9	49	3,589	68.27	3	408,688	26	3,807,916	189.68 ± 47.9	JYIW00000000
*M. oxydans* JX185498	BEL163	Roots of *Salix viminalis* (Lommel Field, Belgium)	28^e^	4/16	44	3,545	68.03	6	164,861	30	3,692,080	153.17 ± 50.4	JYIV00000000
*M. hydrocarbonoxydans* HG941786	SA35	Rhizosphere of *Alyssum serpyllifolium* subsp. *lusitanicum* (Portugal, Samil, Trás-os-Montes)	17^e^	5/11	55	3,663	68.52	3	545,806	10	3,953,585	165.98 ± 38.9	JYJB00000000
*Microbacterium* sp. AB042083	SA39	Rhizosphere of *Alyssum serpyllifolium* subsp. *lusitanicum* (Portugal, Samil, Trás-os-Montes)	40^e^	4/13	50	3,732	68.26	7	232,853	39	3,862,900	94.19 ± 27.4	JXRU00000000
*M. azadirachtae* KF150486	ARN176	Soil (Arnoldstein, Austria)	37	3/9	40	3,910	70.14	7	258,062	40	4,243,255	126.11 ± 36.7	JYIX00000000

a The number after the organism name refers to the 16S rRNA gene NCBI accession number of the closest described relative.

b Number of rRNAs predicted by Barrnap/number of rRNAs estimated based on the coverage of the contigs containing rRNA genes.

c ORFs, open reading frames.

d *L*_50_ and *N*_50_ were calculated using QUAST. *N*_50_ is the contig length such that using longer or equal length contigs produces half (50%) the bases of the assembly. *L*_50_ is the minimal number of contigs that cover ≥50% of the total length.

e Presence of viral sequence confirmed by PHAST.

Genomic DNA was extracted with a phenol-chloroform extraction protocol (16). The Nextera XT kit (Illumina, San Diego, CA) was used for library preparation, and whole-genome sequencing was performed using Illumina MiSeq (MiSeq reagent kit version 3). Raw reads were screened for PhiX contamination using Bowtie2 (17). Adapter and quality trimming were performed in Trimmomatic-0.32 (18). Overlapping reads were subsequently merged using FLASH (19), and long single reads and paired-end reads were assembled with SPAdes 3.1.0 (20). The assembly quality was estimated in QUAST 2.3 (21), and quality control of the mapping data was performed in Qualimap 1.0 (22). PhyloSift version 1.0.1 (23) was used to verify the genome completeness, assessing a list of 40 highly conserved single-copy marker genes, all of which were identified in each assembly. The phylogenetic analysis of the genomes showed no contamination, but a substantial number of viral sequences were detected (10 to 40% of the genome content), which was also confirmed by PHAST (24) for most genomes (Table 1). Moreover, we detected viral sequences in already-published *Microbacterium* sp. genomes in similar percentages.

Genome annotation (summarized in Table 1) was performed in Prokka (25), incorporating Prodigal 2.60, Aragorn, and Barrnap for the prediction of open reading frames (ORFs), tRNAs, and rRNAs, respectively. rRNA detection was confirmed with RNAmmer 1.2 (26). Using antiSMASH 2.0 (27), we detected gene clusters involved in the production of terpenoids in all sequenced strains, except the colorless isolate ARN176. Clusters involved in the production of polyketides and nonribosomal peptides were found in all strains but in DSM 18659 and in DSM 18659 and BEL163, respectively. A deeper comparison of the *Microbacterium* genes involved in HM resistance and mobilization is currently being performed and will be published in a subsequent report.

### Nucleotide sequence accession numbers.

The nucleotide sequences have been deposited at the DDBJ/EMBL/GenBank under the accession numbers provided in Table 1.
